# Technological Properties and Quality Characteristics of Non-Gluten Biscuits Based on Sorghum Flour and Enriched with Sesame and Moringa

**DOI:** 10.3390/foods15091593

**Published:** 2026-05-04

**Authors:** Edwige Bahanla Oboulbiga, Fidèle Wend-bénédo Tapsoba, Ancuţa Petraru, Florin Ursachi, Charles Parkouda, Georgiana Gabriela Codină

**Affiliations:** 1 Food Technology Department (DTA), Institute of Research in Applied Sciences and Technologies (IRSAT), National Center for Scientific and Technological Research (CNRST), Ouagadougou 03 BP 7047, Burkina Faso; obouled@yahoo.fr (E.B.O.); tapfidelew@gmail.com (F.W.-b.T.); cparkouda@yahoo.fr (C.P.); 2 Faculty of Food Engineering, Stefan cel Mare University of Suceava, 720229 Suceava, Romania; ancuta.petraru@fia.usv.ro (A.P.); florin.ursachi@fia.usv.ro (F.U.)

**Keywords:** moringa-enriched sorghum and sesame biscuits, biscuit dough, physical properties, texture, fourier transform infrared spectroscopy, sensory characteristics

## Abstract

The development of gluten-free biscuits with high nutritional value presents a challenge for the food industry. This study evaluated the dough behavior and quality characteristics of gluten-free biscuits obtained using the raw materials sorghum flour, sesame paste, and *Moringa oleifera* leaf powder. Ten formulations were developed, including a control sample without moringa, using a mixture design that generated different combinations between sorghum flour, sesame paste, and powdered moringa. Moringa-enriched biscuits showed significant nutritional improvements, with protein increasing by 40% (12.07–16.93%), fiber by 92% (2.78–5.34%), polyphenols more than twofold (52.88–120.66 mg GAE/100 g), and flavonoids more than threefold (110.44–335.30 mg QE/100 20 g). Technological properties such as rheology, texture, color, and water activity varied with formulation. Moringa addition darkened biscuits (L* 35.61–49.74) and increased hardness by 62% (20.53–33.19 N). All doughs exhibited dominant viscoelastic behavior (G′ > G″), with higher sorghum levels leading to increased viscoelasticity. FTIR analysis confirmed characteristic functional groups of carbohydrates, lipids, and proteins across samples. Sensory evaluation indicated good overall acceptance, with a preference for the control sample and the sorghum-rich formulation (F4), which contained the lowest amounts of sesame paste and powdered moringa. Overall, *Moringa oleifera* enhances both nutritional and technological properties of gluten-free biscuits; therefore, it can contribute to the development of functional products from local resources.

## 1. Introduction

The biscuit takes its name from the word “besquis”, which means “to bake twice”. Originally, it was a wafer that was baked once, then placed in compartments above the oven to be reheated [1]. Today, biscuits are defined as dry products made by baking a dough composed of a mixture of flour, sweeteners, fats, and other food ingredients. Once baked, they retain their organoleptic and commercial qualities for an extended period, up to one year [2].

Biscuits are among the most popular baked goods in the world. They are consumed by all age groups, particularly because of their convenience [3]. Their success is based on several advantages: pleasant taste, ready-to-eat convenience, low cost, nutritional value, availability, and long shelf life due to their very low water activity [2].

The quality of cookies depends on several factors, such as raw materials (quality and quantity), processing steps (mixing, resting, molding, baking, cooling), and sensory criteria such as appearance, color, smell, taste, and uniformity [4].

However, bakery products are facing new challenges related to changing eating habits. Consumers are now looking for products that are safe, tasty, sustainable, and also beneficial to their health [5]. This trend toward healthier eating is pushing the food industry to adopt modern technologies to enrich cookies, both nutritionally and sensorially [6].

Traditionally, the basic ingredients of biscuits are flour, sugar, and fat. Flour is the main ingredient, with starch accounting for 70–75%. Today, many formulations include composite flours or other ingredients to enhance the nutritional value of the biscuits [7,8,9].

Faced with increasing nutritional needs and dependence on soft wheat, researchers have developed various strategies to enrich biscuits with foods rich in micronutrients, fiber, and phytochemicals [8]. Thanks to their widespread consumption, biscuits are an excellent vehicle for combating nutritional deficiencies, particularly through enrichment with micronutrients and bioactive compounds.

Among the alternatives explored, replacing wheat with sorghum makes it possible to produce gluten-free biscuits [10]. In addition, other ingredients such as sesame and moringa are incorporated to enhance nutritional intake. Sesame seeds, for example, are rich in fatty acids, protein, fiber, and vitamin C, and contain essential minerals such as calcium, magnesium, potassium, and phosphorus. They also contain nutraceutical compounds (phenolic compounds, tocopherols) known for their antioxidant properties [11,12].

Moringa, meanwhile, is notable for its high content of beta-carotene; polyphenols; vitamins A, B, and C; and iron and calcium. It is often used as a dietary supplement due to its many nutritional benefits [13,14,15].

This study aimed to determine the physical, technological, sensory, and nutritional properties, as well as the digestibility of proteins in biscuits made from sorghum and enriched with sesame and moringa.

## 2. Materials and Methods

### 2.1. Materials

The plant material used consisted of sesame seeds of the *S42* variety and white sorghum seeds obtained from a supplier in Kaya, North-Central Region (Burkina Faso). The other ingredients, moringa powder, fat (margarine), white powdered sugar, whole powdered milk, fresh egg, baking powder and vanilla sugar, were purchased from a grocery store in Ouagadougou (Burkina Faso).

### 2.2. Experimental Design, Biscuit Formulation and Making Biscuits

The experimental design was generated using Minitab version 18 software based on a mixture design, specifically a simplex lattice design, to evaluate the effect of the proportions of three variable ingredients on the properties of the final product [16]. This experimental approach was supported by previous experimental studies applying mixture designs in bakery products [17,18].

The independent variables studied were sorghum flour (X_1_), sesame paste (X_2_), and moringa powder (X_3_). In this type of design, the sum of the proportions of the mixture components is kept constant. In the present study, the three variable ingredients were constrained to represent 65.5% of the total formulation, while the remaining 34.5% consisted of fixed ingredients, maintained constant across all formulations, namely, powdered sugar (11.8%), margarine (9.1%), powdered milk (4.7%), fresh eggs (4.5%), baking powder (2.5%), vanilla sugar (1.8%), and iodized salt (0.1%).

The proportions of the three variable components were generated by the mixture design and then scaled to represent 65.5% of the total formulation, while the fixed ingredients constituted the remaining fraction. All formulations were expressed on a 100 g total mixture basis, ensuring that the sum of all ingredients equals 100%.

The F0 formulation, composed of 45.80% sorghum flour and 19.70% sesame paste, without the addition of moringa powder (0%), was defined as the reference (control) formulation [19]. It was used solely for comparison purposes and was not involved in defining the factor boundaries, which were independently established based on technological and nutritional considerations.

The factor ranges were defined as follows: 28.45–53.15% for sorghum flour, 9.85–29.55% for sesame paste, and 2.50–7.50% for moringa powder. A total of nine formulations (F1–F9) were generated to cover the entire experimental domain, including points at the vertices, edges, and interior of the mixture space.

Each formulation was prepared under controlled conditions to ensure reproducibility. The control formulation (F0) was used to compare the performance of the formulations obtained from the mixture design.

The simplex mixture design for the biscuit formulations (F1–F9), along with the control sample (F0), is presented in Table 1.

To prepare the dough, the sorghum flour was pre-mixed with the powdered moringa, powdered milk, baking powder and sesame paste on one side, then the powdered sugar, iodized salt, vanilla sugar, fresh egg and water were mixed with a whisk for a few minutes, before the pre-mix (sorghum flour, powdered moringa, powdered milk, baking powder, sesame paste) was gradually incorporated. Kneading was carried out for 10 to 15 min using a BKJ-8 dough mixer, manufactured by Guangzhou Bakestar Machinery Co. Ltd., China, with a 40 L capacity, operating at a mixing speed of 100–158 rpm on the main speed and up to 206–300 rpm on the high speed, to obtain a smooth, elastic, and tender dough. After kneading, the dough was rolled out on a cutting board using a rolling pin until it reached an even thickness of approximately 5–6 mm. The dough was then cut into biscuits of various shapes (round, heart-shaped, rectangular, and star-shaped) using cookie cutters. After shaping, the biscuits were placed on baking sheets and baked in a handmade BBES gas oven (BB Equipments, Ouagadougou, Burkina Faso) with 10 shelves and two burners, preheated to approximately 180 °C, and then baked for 25 to 30 min at a temperature between 145 and 150 °C. After cooling, the biscuits were packaged in plastic bags and stored for analysis.

The dough parameters were evaluated after the final kneading, on 200 g portions. The final dough was placed in standardized containers for measurement. All analyses were performed after mixing before resting or baking. For each parameter, the mean of the three independent measurements was used for statistical analysis.

### 2.3. Determination of the Physical Characteristics of Biscuits

#### 2.3.1. Determination of Thickness and Width

The thickness and width of samples from the ten round biscuit formulations were measured using a caliper, and water activity (aw) was determined using an AquaLab Series 3 analyzer (Decagon Devices, Pullman, WA, USA) at 25 °C [20].

#### 2.3.2. Color Analysis

The color parameters (L*, a*, b*) of the biscuit doughs and baked biscuits were measured using a Minolta chromameter (Model CR-400, Minolta Co., Osaka, Japan). In order to obtain an accurate and consistent color assessment, a CIELab color space was used, where, in terms of colorimetric coordinates, L* represents lightness or luminance while a* represents the green–red color range and b* the blue–yellow color range [21]. Ten readings were recorded for each sample, and the average values were considered.

### 2.4. Textural Properties of Biscuit Dough and Biscuits

The textural properties were evaluated using the TVT 6700 texturometer (Perten Instruments, Hägersten, Sweden) equipped with a 10 kg load cell.

#### 2.4.1. Dough Texture Analysis

Prior to analysis, the dough was shaped into spherical samples of 50 g each. A double compression test (Texture Profile Analysis, TPA) was performed using a cylindrical probe with a diameter of 35 mm. Samples were compressed to 40% of their initial height at a test speed of 5 mm/s, with a trigger force of 20 g. The recovery time between the two compression cycles was 12 s.

The parameters determined for dough were: hardness (N) (maximum force during first compression), adhesiveness (J) (negative work required to detach the probe), stringiness (mm) (distance of filament formation during probe withdrawal), maximum adhesion force (N) (maximum negative force during probe withdrawal), cohesiveness (–) (ratio of work of second to first compression), springiness (–) (ability to recover height between compressions), gumminess (N) (hardness × cohesiveness), and chewiness (N) (gumminess × springiness).

#### 2.4.2. Compression Test of Biscuits

For the compression test, cookies were analyzed using a cylindrical probe with a diameter of 20 mm. The samples were compressed up to 10% of their initial height at a test speed of 2 mm/s, with a trigger force of 10 g and a recovery time of 5 s. The parameter determined from this test was hardness (N).

#### 2.4.3. Fracturability of Biscuits

The fracturability of the cookies was assessed by a single-cycle breaking test (3-point bend rig equipped with a 55 mm aluminum blade). The samples used were circularly shaped (thickness ~4–6 mm, diameter ~30–43 mm). The distance between the support plates was adjusted according to the cookie diameter to ensure proper support. The blade was positioned 2 mm above the sample and moved downward at a speed of 3 mm/s, with a trigger force of 50 g.

The parameters measured were breaking force (maximum force required to break the samples) and fracturability/brittleness (distance until break, mm).

### 2.5. Dynamic Dough Rheological Evaluation

The rheological properties of the dough samples were evaluated using a Thermo-HAAKE MARS 40 rheometer (Karlsruhe, Germany). The frequency sweep test was performed from 1 to 20 Hz in the linear viscoelastic region on samples of laminated cookie dough, which had been left to rest for 30 min at room temperature to eliminate internal deformation. The storage modulus (G′) (elastic property) and loss modulus (G″) (viscous property) were evaluated. Each dough sample prepared at the optimal dough hydration level was analyzed using two parallel plates (d × 40 mm) with a 2 mm gap. During the sweep test, the frequency varied from 0.1 to 20 Hz at a constant strain of 15 Pa. For the temperature sweep test, the dough samples were heated from 25 to 100 °C at a rate of 4 °C per minute.

### 2.6. ATR-FTIR Spectra of Biscuits Analysis

FTIR spectra of the biscuit samples were obtained using a Nicolet iS20 FTIR spectrometer with the Smart iTX (Thermo Scientific Smart iTX, Thermo Fisher Scientific Inc., PORTE, RHEONAUT, Waltham, Germany). The background spectra of the instrument were collected before the samples (0.5 g of each ground biscuit/flour) were mounted on the instrument, and the spectra were recorded with characteristic peaks in wavenumbers from 450 to 4000 cm^−1^ at 16 scans per spectrum

### 2.7. Chemical Composition of the Biscuit Samples

Biscuit composition, including moisture, ash content, crude fat, and crude protein, was determined according to International Association for Cereal Science and Technology (ICC) standard methods, Vienna, Austria: ash content (ICC 104/1), fat content (ICC 136), moisture content (ICC 110/1) and protein content (ICC 105/2). The crude fiber content was determined according to AOAC 962.09 using an automated fiber analyzer (Fibretherm FT12, C. Gerhardt GmbH & Co. KG, Königswinter, Germany), which automates the acid and alkali digestion, filtration, and drying steps. The carbohydrate content was determined by difference, using the equation (Equation (1)) applied by Luca et al. [22].

Carbohydrates (%) = 100 − (fat + protein + ash + moisture)(1)

The energetic value (kcal/100 g) of the biscuit samples was calculated by using the corresponding conversion coefficients according to Equation (2):

Energy (kcal/100 g) = (9 × fat) + (4 × protein) + (4 × Available carbohydrates) +(2 × Fiber)(2)

### 2.8. Total Phenolic Content (TPC), Total Flavonoid Content (TFC) and DPPH Analysis

#### 2.8.1. Extraction of Phenolic Compounds Analysis

The biscuit samples were ground using a Perten 3310 laboratory mill (Perten Instruments AB, Hägersten, Sweden) to obtain flours, which were analyzed for the following chemical characteristics: total polyphenol content (TPC), total flavonoid content (FC) and antioxidant activity (AA) (DPPH radical scavenging capacity). The polyphenol extraction technique was performed as follows: 1 g of biscuit flour was mixed with 5 mL of 40% methanol/acidified water (*v*/*v*, pH = 2, HCl). The samples were then stirred for 15 min using a magnetic stirrer at 60 °C. The extracted samples were filtered through 0.45 µm PTFE membrane filters, collected in 2 mL vials, and stored at −20 °C prior to analysis.

#### 2.8.2. Total Polyphenol Content (TPC) Analysis

A total of 0.1 mL of extract is quantitatively transferred to a test tube, 1 mL of Folin–Ciocâlteu reagent (1:10) and 0.9 mL of Na_2_CO_3_ 7.5% (*w*/*v*) are added. The mixture is stirred and left to stand for 30 min in the dark at room temperature. Next, the total polyphenol content is measured using a 3600 UV-Vis-NIR spectrophotometer (Shimadzu Corporation, Kyoto, Japan) at a wavelength of 750 nm, based on a calibration curve of a known polyphenol (gallic acid). To obtain the calibration curve, gallic acid solutions with concentrations of 10, 25, 50, 100, 250, and 500 mg/L were used. The calibration curve showed a high degree of linearity (R^2^ = 0.9966).

#### 2.8.3. Total Flavonoid Content Analysis

From the prepared extract, as presented in Section 2.3, 1 mL of solution was used and mixed with 60 µL of 5% NaNO_2_ (*w*/*v*). The mixture was left to stand for 5 min, and then 60 µL of AlCl_3_ (5% *w*/*v*) was added. After an additional 6 min, 0.4 mL of 1 N NaOH was added. Then, after a further 10 min of standing in the dark, the absorbance was read for each sample at a wavelength of 510 nm using an HR4000CG-UV-NIR spectrometer, Largo, FL, USA. To obtain the calibration curve, quercetin solutions with concentrations of 50, 100, 150, 200, 250, and 300 mg/L were used. The calibration curves showed a high degree of linearity (R^2^ > 0.99). The concentrations determined from the quercetin calibration curve are expressed in mg QE/L and then converted to mg QE/kg.

#### 2.8.4. Antioxidant Capacity (DPPH) Analysis

The determination of 1,1-diphenyl-2-picrylhydrazyl (DPPH) radical scavenging activity required the following sample preparation: 315 µL of whole wheat flour extract was mixed with 2.25 mL of 2,2-diphenyl-1-picrylhydrazyl (DPPH) solution, freshly prepared at a concentration of 80 µM. The mixture was left to stand at room temperature in the dark for 30 min. Next, the ability of the prepared extract to scavenge stable DPPH radicals was measured using a UV–Vis–NIR 3600 spectrophotometer (Shimadzu Corporation, Kyoto, Japan) at a wavelength of 515 nm. The DPPH radical scavenging activity was expressed as antioxidant capacity (AA%) and was calculated using Equation (3), where A_0_ is the absorbance of the control sample and A is the absorbance of the analyzed sample.

AA% = (1 − A/A_0_) × 100 (3)

### 2.9. In Vitro Protein Digestibility

Enzymatic digestion of proteins was performed according to the method described by Minekus et al. [23]. This method simulates digestion, typically including the oral, gastric, and small intestine phases. It mimics physiological conditions in vivo, considering the presence of digestive enzymes and their concentrations, pH, digestion time, and iodized salt concentrations, among other factors.

Oral phase: In a 50 mL Falcone tube, 5 g of raw cashew nut meal was mixed with 4 mL of the FSS + α-amylase solution, the preparation of which is described in Table 1. To this mixture, 25 μL of 0.3 M CaCl_2_ and 975 μL of Milli-Q water were added, and the whole mixture was incubated for 2 min with stirring.

Gastric phase: During this phase, 8 mL of the FGS stock solution + pepsin was added to the mixture previously obtained in the oral phase. Then, 5 μL of CaCl_2_ (0.3 M) was added and the pH of the mixture was adjusted to 3 using a HCl solution. The volume was made up to 20 mL with Milli-Q water and the mixture was incubated at 37 °C with agitation for 2 h.

Intestinal phase: During the intestinal phase, 10 mL of the 10 mM bile salt solution was added to the 20 mL food bolus. Next, 6 mL of the FIS solution + 200 U/mL pancreatic α-amylase was added. The pH was adjusted to 7 using NaOH, the volume of which was noted. Next, 40 μL of CaCl_2_ (0.3 M) was added, followed by 1 mL of the 100 U/mL porcine trypsin solution and 1 mL of the 25 U/mL bovine chymotrypsin solution. The volume was made up to 40 mL with Milli-Q water and incubated at 37 °C with agitation for 2 h. After incubation, the tubes were centrifuged for 30 min at 4 °C and 13,000 rpm in a refrigerated centrifuge, then 20 mL of the supernatant from each sample was taken for protein measurement. Protein bioaccessibility was calculated using the following method: Protein bioaccessibility (%) = Protein content after digestion/Protein content before digestion × 100.

### 2.10. Sensory Evaluation

Sensory analysis was conducted at the Sensory Analysis Laboratory of the Department of Food Technology (DTA). A panel of 30 semi-trained panelists, comprising men and women with experience in sensory analysis, took part in this study. The biscuits were assessed 24 h after baking using a hedonic test on a five-point scale, ranging from 1 (“dislike very much”) to 5 (“like very much”). The evaluated characteristics were appearance, flavor, taste, texture and overall acceptability.

During each session, four samples (the three best formulations and the control sample), identified by numerical codes, were randomly presented to panelists on plastic plates in individual booths equipped with evaluation sheets, pens, and water for rinsing between evaluations. Sensory analysis was conducted at room temperature (approximately 25 °C). This study was approved by the Health Research Ethics Committee (No. 2024-04-102), and all participants provided informed consent.

### 2.11. Statistical Analysis

The data were analyzed using descriptive statistics (means, standard deviations) using Excel version 2016 software. The analysis of variance (ANOVA) was performed using the XLSTAT software (Ver. 2016) at a Tukey’s test significance level of 5%. The experimental design used for the formulation trials was an extreme vertices design, generated using Minitab version 18.1. (Minitab Inc., State College, PA, USA).

### 2.12. Ethical Approval and Recruitment of Participants

This study was approved on 8 April 2024 by the Ethics Committee of the Ministry of Health of Burkina Faso, clearance number 2024-04-102. The study participants for the sensory test were drawn from a trained panel of professionals in the production and analysis of biscuits, staff from the Food Technology Department and students from Burkina Faso universities, all of whom were experienced in carrying out sensory analyses. All methods were carried out in accordance with the sensory analysis protocol according to the test type. Informed consent was obtained from all participants (age ≥ 18 years) involved in this study.

## 3. Results and Discussion

### 3.1. Physical Characteristics and Textural Characteristics of the Biscuit Doughs

The colors and textural characteristics of the biscuit doughs before baking are shown in Table 2. The luminance (L*) of the biscuit dough of the F0 formulation without moringa powder (L*: 56.82) was lighter than the other biscuit dough formulations, as the L* dimension indicates the brightness: 100 for white and 0 for black. The value of the color parameter a* of the biscuit doughs indicates redness when positive and greenness when negative. All biscuit doughs except F0 showed varying degrees of greenness. The a* value (−4.68) of F4 biscuit doughs was the lowest, indicating more pronounced greenness than the others. Also, all biscuit doughs showed a yellow color to different degrees, as b* indicates yellow color when positive. F4 biscuit doughs (b*: 26.75) exhibited the highest yellow value among the biscuits. A significant difference was observed (*p* < 0.05) for the L*, a* and b* parameter values. In general, F0 biscuit doughs without moringa powder were lighter and redder than the color of the other biscuit doughs. This difference can be explained by the presence of moringa powder in the other biscuits. The green color of Moringa is attributed to the presence of phenolic compounds that influence its pigmentation [24,25]. 

The textural parameters of the different formulations (F0–F9) showed variations depending on their composition. Hardness differed significantly among samples (*p* < 0.05), with values ranging from 8.44 N (F2) to 18.97 N (F8). Formulations F1 (18.16 N) and F4 (17.43 N) also exhibited relatively high values, whereas the remaining samples showed intermediate values with no significant differences.

Adhesiveness ranged from −1.67 to −2.89 N, with the highest value observed for F3, indicating a more adhesive dough; however, no significant differences were detected among the formulations (*p* > 0.05). Adhesive energy varied from −172.29 J (F1) to −376.94 J (F3), also without statistically significant differences. This behavior may be explained by interactions between lipids and dietary fibers present in sesame and moringa, which can influence water retention and the surface properties of dough systems [26]. Gumminess followed a trend similar to hardness, with the highest value recorded for F8 (3.15 N) and the lowest for F2 (1.30 N) (*p* < 0.05). These variations may be attributed to the structuring effect of sorghum starch as well as the proteins and fibers from moringa. Conversely, sesame lipids may weaken starch–protein interactions, resulting in a softer texture [27,28,29].

Gumminess followed a trend similar to hardness, with the highest value recorded for F8 (3.15 N) and the lowest for F2 (1.30 N) (*p* < 0.05). These variations may be attributed to the structuring effect of sorghum starch as well as the proteins and fibers from moringa. Conversely, sesame lipids may weaken starch–protein interactions, resulting in a softer texture. Chewiness also ranged from 1.30 N (F2) to 3.15 N (F8), with significant differences observed among the formulations (*p* < 0.05). In contrast, springiness remained nearly constant, around 1.00 for all formulations, with no significant differences, indicating a similar recovery capacity after compression. Cohesiveness varied slightly between 0.14 and 0.21, without statistically significant differences (*p* > 0.05). Likewise, adhesiveness did not show significant variation among the samples, suggesting that the elasticity and internal bonding of the dough matrix remained relatively stable across formulations [30,31].

Overall, the formulation mainly affected hardness, gumminess, and chewiness, whereas springiness and cohesiveness remained relatively stable. This suggests that formulation changes influenced the overall mechanical resistance of the dough more than its fundamental viscoelastic structure.

### 3.2. Physical Characteristics of the Biscuit Samples

**Figure 1:**
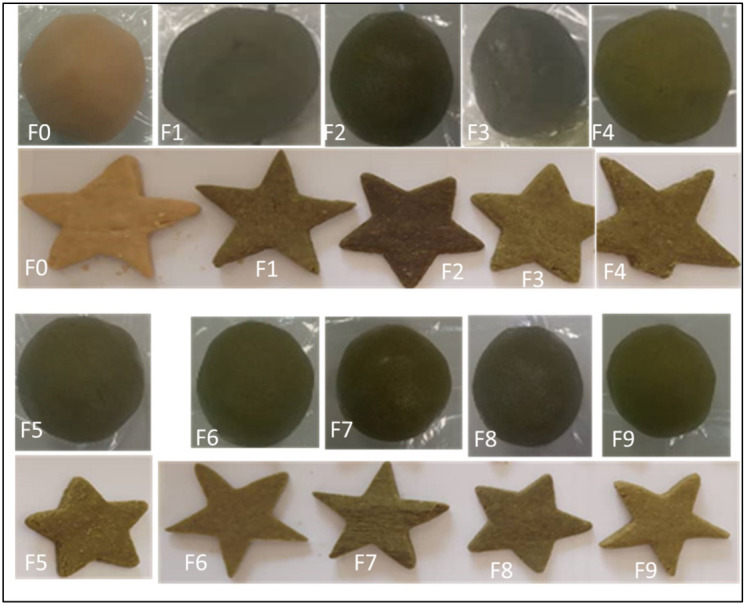
Images of biscuit dough and biscuits after baking of the different formulations F0, F1, F2, F3, F4, F5, F6, F7, F8, F9.

The data of color (L*, a* and b*), physical properties (thickness, width) and water activity of the biscuit samples after baking are presented in Table 3. Images of the dough and biscuit samples are shown in Figure 1. The values of color parameters, thickness, width and water activity showed a significant difference (*p* < 0.05) between the biscuit formulations. The L*, a* and b* parameters of the biscuits showed a darker, greener and more yellow appearance respectively. Formulations F8 and F9 showed a greener coloration, consistent with negative a* values, while F1 had a virtually neutral hue. This could be due to the higher amount of moringa powder incorporated into the biscuit recipe. The phenolic content of the biscuits could decrease the brightness of the biscuits with an increase in the formation of melanoidin, resulting in a darkening of the product [32].

The variations in thickness and width (diameter) observed between the different cookie formulations are directly related to the functional properties of the ingredients incorporated, particularly sorghum flour, *Moringa oleifera* powder, and sesame paste [33]. These dimensional parameters are important technological indicators of the behavior of the dough during baking, particularly the spread ratio. The incorporation of sorghum flour tends to increase the thickness of the cookies while reducing their width. This change can be explained by the high starch and fiber content of sorghum, which helps to strengthen the structure of the dough and limit its spread during baking. Several studies have shown that the addition of sorghum flour significantly influences the physical characteristics of cookies, including their diameter, thickness, and texture [10,28]. Similarly, the addition of *Moringa oleifera* powder, due to its high fiber and protein content, increases the consistency of the dough and reduces its expansion in the oven. This generally results in thicker cookies with a smaller diameter compared to control formulations. Previous studies have confirmed that moringa powder supplementation significantly alters the physical parameters of cookies, particularly thickness and diameter [34].

Conversely, sesame enrichment, particularly in the form of a high-fat paste, increases the diameter of cookies and reduces their thickness. Fat reduces the apparent viscosity of the dough and weakens the cohesion of the structural network, thereby facilitating spreading during baking. This increase in the spreading factor results in wider and thinner cookies, as reported in several studies on sesame-enriched biscuits [35]. Thus, the final thickness and diameter of the cookies result from a balance between the structuring effects of fiber- and protein-rich ingredients (sorghum and moringa), which limit spreading, and the plasticizing effect of sesame lipids, which accentuate it.

The results show that water activity varies significantly between samples. Samples F2, F1, and F8 have the highest aw values (0.32–0.36) and are significantly higher (*p* < 0.05) than F0, F4, and F6, which have the lowest values (0.17–0.20). The other samples (F3, F5, F7, F9) have intermediate values and differ significantly from some extreme groups, but not all, highlighting a significant variation in aw depending on the formulation of the samples. The water activity range of 0.11–0.36 was lower than 0.6, indicating better cookie stability during storage. This margin corresponds to the minimum water activity for the growth of microorganisms. Low water activity was obtained for the F4 sample, which presented a low amount of moringa powder (2.5%). This could be due to the high fiber and polyphenol content of moringa, which can absorb more water and thus reduce the water activity of biscuits.

### 3.3. Textural Characteristics of Biscuit Formulation

The textural characteristics of biscuit formulations are presented in Table 4. Texture represents a set of physical and geometric properties that influence consumer perception of the food product. In the food industry, texture is a key attribute used to assess overall product quality and acceptability [36]. In baked products such as biscuits, hardness and fracturability are considered important indicators of quality, as they describe resistance to deformation and the tendency to break under applied force.

Hardness values ranged from 20.53 N to 33.19 N, with the highest values observed in samples F8 and F5. This increase in hardness can be attributed to the incorporation of moringa powder, which is rich in dietary fiber and exhibits high water absorption capacity. Fiber-rich ingredients can increase the structural rigidity and density of baked products, leading to higher resistance to deformation [37]. However, excessively high hardness may reduce crispness and negatively affect sensory perception, while very low hardness may result in a fragile and overly brittle structure [4].

Breaking force values varied between 0.53 N and 1.50 N, while fracturability ranged from 0.64 mm to 2.35 mm. These relatively low fracturability values indicate that the biscuits exhibited a brittle structure, breaking at small deformation distances. This behavior is characteristic of low-moisture baked products, where fracture dominates over elastic deformation.

Fracturability showed significant variations (*p* < 0.05) between the biscuit formulations. Sample F2 exhibited the highest fracturability (2.35 mm), suggesting a slightly less brittle structure compared to the other formulations, while samples such as F4, F8, and F9 fractured at shorter distances, indicating increased brittleness.

From a sensory perspective, the breaking behavior of biscuits plays an important role in flavor perception. Effective fracture during mastication increases the surface area available for interaction with saliva, promoting the release of aromatic compounds and enhancing flavor perception. Conversely, limited fragmentation may reduce this effect and diminish perceived flavor intensity [38].

### 3.4. Dynamic Dough Rheological Properties of Biscuits

**Figure 2:**
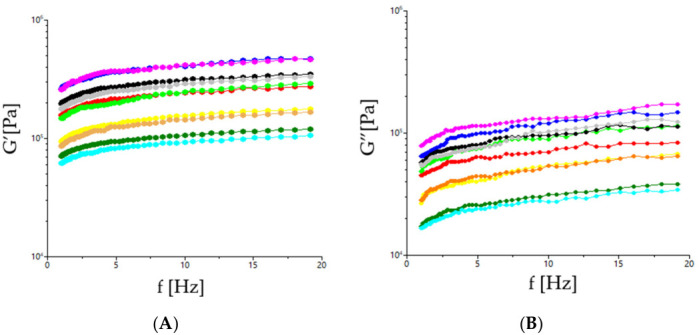
Evaluation with frequency of storage modulus (**A**) (G′—open symbols) and loss modulus (**B**) (G″—solid symbols) for dough samples (-●-F0; -●-F1; -●-F2; -●-F3; -●-F4; -●-F5; -●-F6; -●-F7; -●-F8; -●-F9).

**Figure 3:**
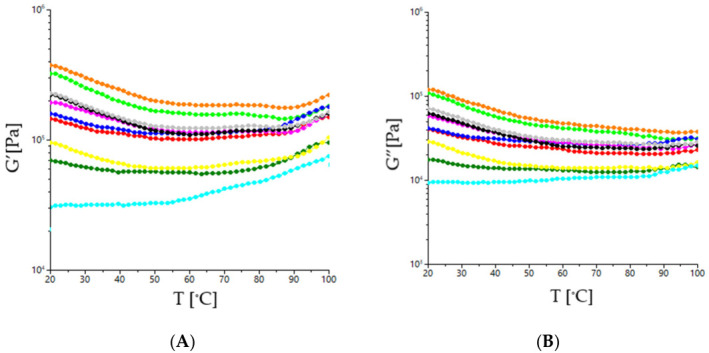
Evaluation with temperature of storage modulus (**A**) (G′—open symbols) and loss modulus (**B**) (G″—solid symbols) for dough samples (-●-F0; -●-F1; -●-F2; -●-F3; -●-F4; -●-F5; -●-F6; -●-F7; -●-F8; -●-F9).

The dynamic dough rheological properties with different mixes of sorghum, moringa, and sesame are shown in Figure 2 and Figure 3. Figure 2 presents the frequency sweep results for dough mixes, whereas Figure 3A,B present the evolution of the dynamic moduli G′ and G″ with temperature. As may be seen from Figure 2, both the storage (G′) and loss (G″) moduli increased with increasing frequency. The G′′ modulus was lower than the G″ one which shows a solid-like behavior for all the dough samples [39,40]. Moduli values vary with the increasing frequency and are significantly different depending on the amounts of sorghum, sesame and moringa used in the dough recipe. Higher amounts of sorghum led to higher values for G′ and G″ (samples F4, F5), which indicates higher viscoelasticity probably due to the ability of starch to form a more stable three-dimensional network [41]. The dough samples with higher amounts of sesame led to lower values for the dynamic moduli G′ and G″ but higher ones for tan δ, highlighting a more viscous behavior and a less elastic one. This behavior may be due to the adsorption of fats onto the surface of protein globules and starch granules, which causes their hydrophobicity, accompanied by a reduction in their water-binding capacity and a slowing down of their hydration [42]. Similarly, moringa, through its high protein and fiber content, increased dough elasticity, but higher amounts in the dough recipe led to lower G′ and G″ values, indicating a softer and more crumbly dough.

The effect of increasing temperature on the rheological properties of dough indicates a decrease in the dynamic moduli G′ and G″ values, leading to lower dough stiffness. This may be due to the fact that as the temperature of the dough increases, the speed of the swelling and peptization processes of the flour colloids increases, the amount of bound water decreases, and the liquid phase of the dough increases and, as a result, its elasticity and viscosity decrease [43]. Also, the decrease in dough elasticity and viscosity may be attributed to the intensification of enzymatic activity from the dough system with the increasing temperature [44]. Moreover, the sugar and fat amount from the dough recipe tends to decrease the G′ and G″ moduli due to the fact that sugar acts as a plasticizer through a dehydration action exerted on the flour components, while lipids coat starch and proteins from the dough system, which may cause their hydrophobicity [45]. This will reduce starch and protein ability to bind water, slowing down their hydration and leading to a decrease in dough visco-elasticity [46]. Dough samples F8, F3 and F7 presented the best visco-elasticity, probably due to the optimal balance between starch, protein and fiber, which maintains dough stability during heating. Around 70 °C, the storage (*G′*) and loss (*G″*) moduli begin to increase slightly, probably due to the starch gelatinization process. This increase tends to be higher for the samples with high amounts of sorghum in their recipe, which contain more starch and low sesame seed, which contain more lipids such as F4. Also, a high amount of moringa increased the dynamic moduli of sorghum dough during heating due to its strong water-binding capacity and filler effect, which reinforced the gelatinized starch matrix and limited granule disintegration, resulting in a more stable viscoelastic structure [47].

### 3.5. ATR-FTIR Spectra of the Biscuits

**Figure 4:**
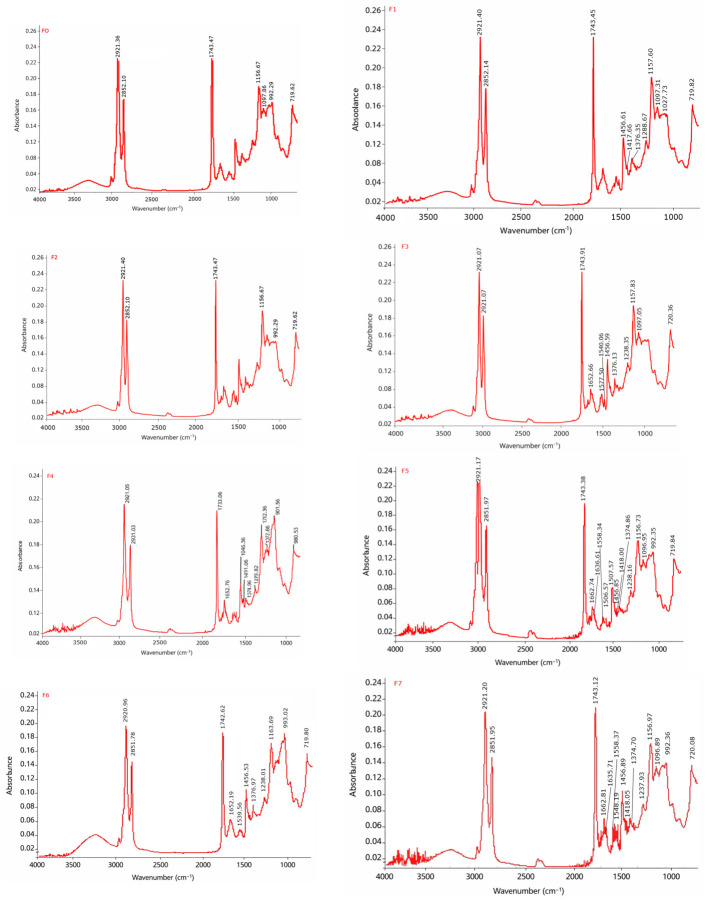

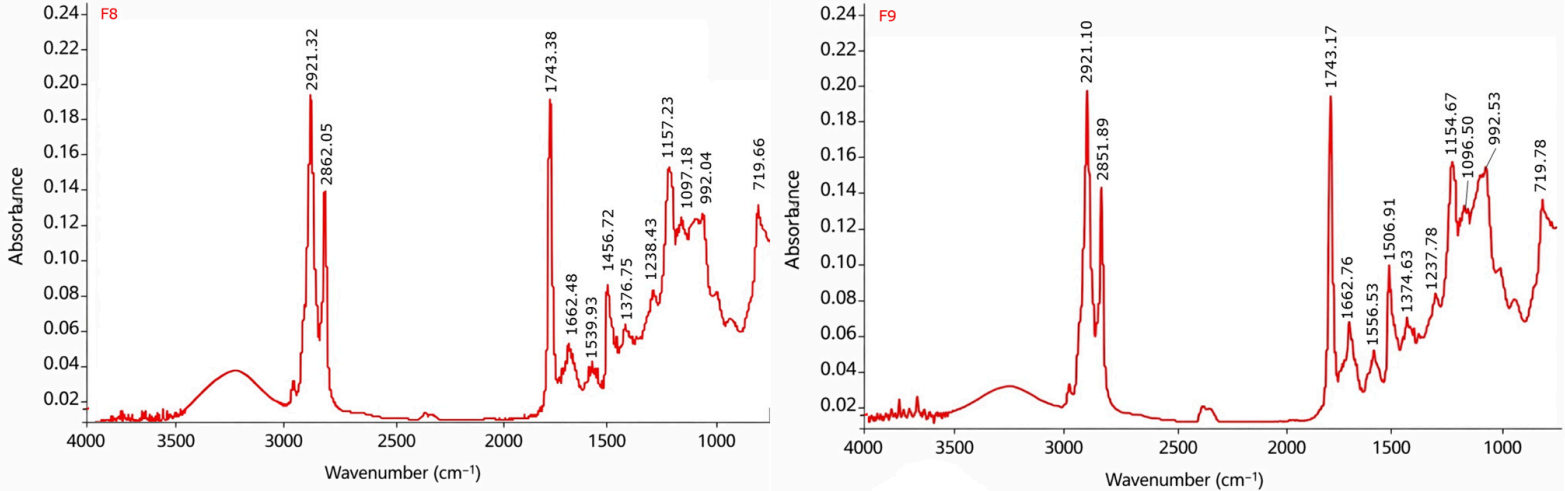
Fourier transform infrared spectroscopic (FTIR) spectra of biscuit samples.

The FTIR spectra of the biscuit samples ranged from 700 to 4000 cm^−1^ (Figure 4). The FTIR spectra exhibited similar characteristic peaks with variations in intensity.

The peaks of the biscuit samples observed at 3290 and 3000 cm^−1^ correspond to O-H (alcohol, phenols) groups characterizing carbohydrate molecules, while the peaks at 2850 and 2920 cm^−1^ indicate the alkyl (methylene) and N-H (ammonium ions) bonds, suggesting the presence of proteins. Also, the peak found at 1650 cm^−1^ was attributed to the C=O stretching vibration characteristic of the protein (amide) structure. The biscuit samples peak at 1743 cm^−1^ was attributed to the C=O stretching vibration corresponding to fats. The biscuit sample peaks of 1456 cm^−1^ indicate C-C (aromatic) stretching vibration. The peak at 1150 cm^−1^ is due to etheric C-O stretching vibrations (esters, alcohols) related to carbohydrate structures. The peak at 719 cm^−1^ indicates a stretching vibration of the C-H bond of aromatic compounds [48]. Peaks at 992 cm^−1^ and 990 cm^−1^ are attributed to the stretching vibration of the C-H vinyl bond of monosubstituted alkenes [49].

Similar results were reported by Nicy et al. and Adebiyi [50,51] with chocolate cookies incorporated with surimi powder and millet flour. Zarroug et al. [52] also reported similar spectral wavenumbers in cookies enriched with *Zizyphus lotus* L. In all biscuit samples, major peaks were observed at 3500 cm^−1^, 2800 (O-H stretching vibrations), 2943 cm^−1^ (C-H stretching vibration), 1645 cm^−1^ and 1742 cm^−1^ (C-O stretching vibration of the α and β-unsaturated compound respectively).

The obtained spectra showed the presence of carbohydrates, proteins and fat in the studied cookies. Peaks caused by structural carbohydrate, protein and fat molecules were also found in diet cookies by Karaca Açari [53]. The presence of alkyl-related bands suggests the contribution of amino acid side chains, including sulfanyl (cysteine) and amino (lysine) groups.

### 3.6. Chemical Composition of Biscuit Samples

The chemical composition and energy value of the biscuit formulations are shown in Table 5. The moisture, ash, fat, protein, carbohydrate, and fiber contents varied depending on the formulation. Moisture ranged from 4.05 (F0, F1) to 4.13 (F4, F9), ash between 3.33 (F4) and 4.06 (F3), fat between 17.68 (F1) and 31.95 (F7), protein between 12.07 (F6) and 16.93 (F8), carbohydrates between 43.77 (F3) and 61.23 (F1), and fiber between 2.12 (F4) and 5.34 (F3). With the exception of water and fiber content, significant differences (*p* < 0.05) were observed between the different biscuit samples for ash, lipids, protein, and total sugar content. Some moringa-enriched biscuits had a particularly high macronutrient content.

The moisture values obtained are in line with recommendations for optimal storage, limiting microbial growth and chemical changes, which allows for a longer shelf life. In addition, lipids play an essential role in the textural and organoleptic properties of biscuits after baking [54]. Some moringa-enriched biscuits had higher macronutrient contents compared to non-enriched cookies. These results are consistent with those reported by Ifediba et al. [55], who observed an increase in macronutrients following the addition of *Moringa oleifera* leaf powder. A similar trend has been reported in most studies on the incorporation of edible plant powders such as moringa into biscuits [9,56]. Zarroug et al. [57] showed that the addition of *Vicia narbonensis* seed flour improves the protein and ash content of the formulated cookies.

The energy value of the cookies ranged from 466 kcal/100 g for formulation F4 to 516 kcal/100 g for formulation F3. A significant difference (*p* < 0.05) was observed between the different biscuit samples. The biscuits with the highest fat content had a higher energy value, indicating a proportional correlation between lipid content and energy value. With the exception of samples F2, F3, F7, and F8, all moringa-enriched biscuits had a lower energy value than the unenriched control (F0). This difference can be explained by variations in the composition of the formulations, particularly the fat and carbohydrate content, which directly influence the energy contribution of the biscuits. However, the addition of moringa powder did not result in a significant reduction in the energy value of the enriched biscuits.

### 3.7. Total Phenolic Content (TPC), Total Flavonoid Content (TFC) and DPPH

The bioactive compounds of the biscuits are shown in Table 6. The total polyphenol content (TPC), total flavonoid content and antioxidant activity (DPPH) ranged from 52.88 mg/100 g (F0) to 120.66 mg/100 g (F9); from 96.18 mg/100 g (F4) to 335.30 mg/100 g (F7); and from 6.88% (F9) to 8.06% (F3), respectively.

The results showed significant differences (*p* < 0.05) between the different biscuit formulations. All formulations showed high antioxidant activity, which can be attributed to the presence of bioactive compounds in *Moringa oleifera* powder and sesame seeds. Several studies have demonstrated that M. oleifera is rich in polyphenols and flavonoids with strong antioxidant capacity, capable of scavenging free radicals and inhibiting oxidation in various plant extracts [58]. Similarly, sesame seeds possess significant antioxidant activity correlated with their content of phenolic compounds and flavonoids, including lignans such as sesamin and sesamolin, which contribute to free radical scavenging capacity [58].

### 3.8. In Vitro Protein Digestibility

**Figure 5:**
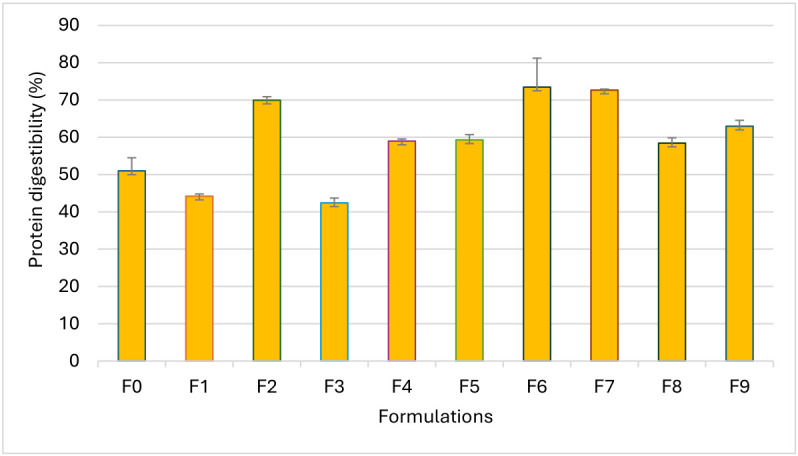
In vitro protein digestibility of biscuit samples.

The protein digestibility of the biscuit samples is shown in Figure 5. The results showed that protein digestibility is significantly different (*p* < 0.05) depending on the biscuit formulations. Among these, sample F6 has the highest protein digestibility (73.48%), followed by samples F7, F2, F9, F8, F5, and F4. Biscuit samples F3, F1, and F0 had the lowest values, at 42.42%, 44.21%, and 51.00%, respectively. High protein digestibility suggests that biscuits obtained from sorghum, sesame, and moringa allow the body to absorb protein efficiently. The addition of moringa powder significantly improves protein digestibility. The digestibility of proteins in sorghum-based products can be significantly improved by enriching them with *Moringa oleifera* powder, which increases the accessibility of proteins to digestive enzymes. A study by Mafukata et al. [8] showed that adding moringa leaf powder to a fermented sorghum product increases in vitro protein digestibility from around 50% to over 68–70%, mainly due to the reduction in antinutritional factors. Other studies indicate that moringa and sesame proteins have high digestibility profiles and that combining sorghum with these functional ingredients significantly improves the nutritional value of the finished product [59,60]. Other research indicates that optimal digestion is promoted by combining several sources of plant protein in order to benefit from all the essential amino acids necessary for protein synthesis. By combining different sources of plant protein in the same food or during meals, it is possible to obtain a more complete essential amino acid profile than with a single source. Optimized blends can improve protein quality scores measured by indicators such as DIAAS and reproduce profiles close to those of animal proteins, thus ensuring effective protein nutrition and promoting protein synthesis in the body [8]. In addition, factors related to the food matrix including the structure of the food and the technological processes influence digestibility due to the presence or absence of elements that interfere with digestion [61].

### 3.9. Sensory Characteristics of Biscuits

**Figure 6:**
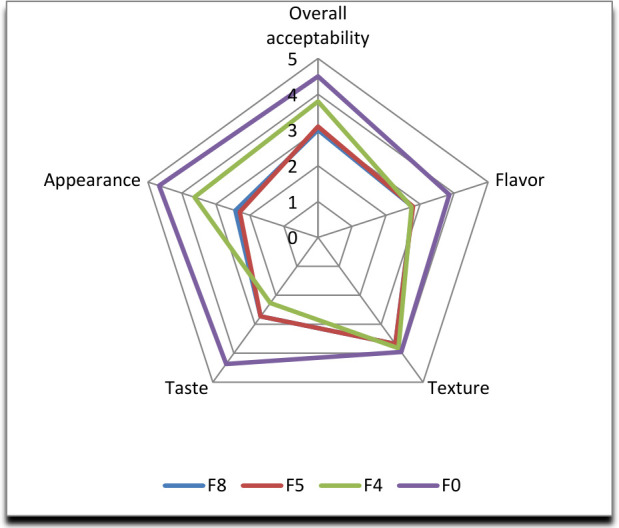
Sensory characteristics of the four highest-ranked biscuit samples.

The sensory evaluation results, expressed as hedonic mean values on a 1–5 scale, showed significant differences among the biscuit samples analyzed (Figure 6). Formulation F0 exhibited the highest scores for most sensory attributes, including appearance (4.67), flavor (4.60), taste (4.37) and overall acceptability (4.50). These results indicate strong panelist appreciation and reflect superior overall sensory quality. This evaluation can be attributed to an optimized balance of organoleptic components, particularly those related to flavor. Formulation F4 ranked second, showing satisfactory scores for appearance (3.63), aroma (3.27), texture (3.83), and overall acceptability (3.80). However, lower values for taste (2.27) and overall flavor (2.75) suggest an imbalance in the sensory profile, especially regarding sweetness perception, which may limit its overall acceptability. Formulations F5 and F8 exhibited comparable sensory profiles, characterized by intermediate to low scores for most sensory characteristics analyzed. Their overall acceptability values (3.10 and 3.00, respectively) indicate a generally neutral perception by the panelists. Although texture scores were relatively uniform (3.68), these formulations were penalized for lower performance in appearance, aroma, and flavor. Moreover, texture scores showed low variation among the formulations (3.68–3.96), suggesting that this sensory characteristic was less discriminating in the overall product evaluation. In contrast, flavor-related attributes appeared to be the main determinants of overall sensory acceptability. This analysis highlights the best sensory appreciation for formulation F0, followed by F4, while F5 and F8 exhibited lower sensory performance. These results confirm that optimizing flavor and appearance characteristics is a key strategy for improving the overall acceptability of the product. These results indicate that the incorporation of moringa influences the sensory acceptability of the biscuits, with the effect depending on the incorporation rate. Dabo et al. [19] found low levels of unpleasantness in cereal biscuits with sesame.

## 4. Conclusions

The incorporation of moringa and sesame powder into sorghum-based biscuits significantly influenced the physicochemical, rheological, textural, and sensory properties of the formulated products. The results showed that F0 doughs and biscuits without moringa were lighter in color and redder, while the addition of moringa significantly (*p* < 0.05) altered the parameters of firmness, maximum adhesion force, gumminess, and chewiness. The F8 dough was distinguished by a firmer and more gummy texture, while F3 was more adhesive. For the biscuits, the fracture, elasticity, and cohesion parameters also varied significantly depending on the formulations, confirming the direct influence of moringa and sesame levels on textural quality. FTIR analysis revealed the presence of major nutritional compounds (carbohydrates, proteins, and lipids), attesting to the nutritional richness of the enriched biscuits. The increase in storage (G′) and loss (G″) moduli with frequency indicates enhanced viscoelastic behavior of the doughs. In terms of sensory properties, formulation F4 was the most appreciated, combining a good nutritional balance (high total lipids and sugars) with satisfactory organoleptic characteristics.

Enriching sorghum- and sesame-based biscuits with moringa improves their nutritional value while maintaining satisfactory sensory acceptability, particularly for formulation F4. These cookies are therefore a promising functional alternative, particularly suitable for people with gluten intolerance.

## Figures and Tables

**Table 1:** Simplex mixture design (simplex lattice) illustrating the proportions of variable ingredients (X_1_–X_3_) for formulations F1–F9 and the control F0.

Samples	Sorghum Flour X_1_ (%)	Sesame Paste X_2_ (%)	Powdered Moringa X_3_ (%)	Total Variables (%)
F0	45.80	19.70	0.00	65.50
F1	48.15	9.85	7.50	65.50
F2	33.45	29.55	2.50	65.50
F3	28.45	29.55	7.50	65.50
F4	53.15	9.85	2.50	65.50
F5	40.80	19.70	5.00	65.50
F6	44.48	14.78	6.25	65.50
F7	37.13	24.63	3.75	65.50
F8	34.63	24.63	6.25	65.50
F9	46.98	14.78	3.75	65.50

**Table 2:** Textural characteristics and color parameters of biscuit doughs.

Formulations	Hardness (N)	Maximum Adhesion Force (N)	Adhesiveness (J)	Gumminess (N)	Chewiness (N)	Springiness (Dimensionless)	Cohesiveness (Dimensionless)	L*	a*	b*
F0	13.31 ± 1.90 ^ab^	−1.34 ± 0.03 ^a^	−269.90 ± 2.82 ^a^	1.86 ± 0.87 ^ab^	1.86 ± 0.71 ^ab^	1.00 ± 0.09 ^a^	0.21 ± 0.09 ^a^	56.82 ± 0.21 ^a^	6.20 ± 0.15 ^a^	25.11 ± 0.10 ^abc^
F1	18.16± 1.97 ^a^	−1.94 ± 0.06 ^ab^	−172.29 ± 2.40 ^a^	2.78 ± 0.70 ^a^	2.78 ± 0.71 ^a^	1.00 ± 0.00 ^a^	0.15 ± 0.00 ^a^	35.74 ± 0.11 ^c^	−4.48 ± 0.25 ^e^	21.88 ± 0.38 ^abcd^
F2	8.44 ± 0.89 ^b^	−1.67 ± 0.04 ^ab^	−243.47 ± 1.68 ^a^	1.30 ± 0.74 ^b^	1.30 ± 0.60 ^b^	1.00 ± 0.00 ^a^	0.16 ± 0.00 ^a^	36.35 ± 0.05 ^c^	−2.00 ± 0.58 ^bc^	20.84 ± 0.89 ^bcde^
F3	15.92 ± 1.81 ^ab^	−2.89 ± 0.09 ^b^	−376.94 ± 2.44 ^a^	2.62 ± 0.60 ^a^	2.62 ± 0.60 ^a^	1.00 ± 0.01 ^a^	0.17 ± 0.01 ^a^	28.25 ± 0.20 ^g^	−1.22 ± 0.23 ^b^	12.11 ± 0.41 ^f^
F4	17.43 ± 1.83 ^a^	−1.92 ± 0.07 ^ab^	−202.93 ± 1.61 ^a^	2.48 ± 0.68 ^ab^	2.48 ± 0.68 ^ab^	1.00 ± 0.00 ^a^	0.14 ± 0.00 ^a^	39.72 ± 0.62 ^b^	−4.68 ± 0.04 ^e^	26.75 ± 0.02 ^a^
F5	12.91 ± 1.44 ^ab^	−1.98 ± 0.02 ^ab^	−223.57 ± 3.76 ^a^	1.93 ± 1.14 ^ab^	1.93 ± 0.86 ^ab^	1.00 ± 0.01 ^a^	0.15 ± 0.01 ^a^	34.06 ± 0.06 ^de^	−3.67 ± 0.06 ^de^	20.08 ± 0.34 ^cde^
F6	16.17 ± 1.21 ^ab^	−1.96 ± 0.03 ^ab^	−194.66 ± 3.28 ^a^	2.49 ± 0.51 ^ab^	2.49 ± 0.51 ^ab^	1.00 ± 0.00 ^a^	0.15 ± 0.00 ^a^	35.23 ± 0.25 ^cd^	−4.36 ± 0.02 ^e^	20.75 ± 0.37 ^bcde^
F7	13.00 ± 1.50 ^ab^	−2.11 ± 0.04 ^ab^	−235.46 ± 1.90 ^a^	1.97 ± 0.86 ^ab^	1.97 ± 0.86 ^ab^	1.00 ± 0.00 ^a^	0.15 ± 0.00 ^a^	33.18 ± 0.17 ^e^	−2.70 ± 0.19 ^cd^	19.38 ± 0.08 ^de^
F8	18.97 ± 1.68 ^a^	−2.80 ± 0.05 ^ab^	−311.65 ± 2.73 ^a^	3.15 ± 0.81 ^a^	3.15 ± 0.71 ^a^	1.00 ± 0.00 ^a^	0.17 ± 0.00 ^a^	30.31 ± 0.17 ^f^	−2.82 ± 0.18 ^cd^	15.94± 0.43 ^ef^
F9	16.26 ± 1.07 ^ab^	−1.93 ± 0.07 ^ab^	−180.12 ± 1.07 ^a^	2.38 ± 0.34 ^ab^	2.38 ± 0.21 ^ab^	1.00 ± 0.00 ^a^	0.15 ± 0.00 ^a^	39.52 ±0.21 ^b^	−4.66 ± 0.89 ^e^	25.42 ± 0.05 ^ab^

The values are expressed as the mean ± standard deviation. Different superscript letters after values mean significant difference (ANOVA, Tukey’s test, *p* < 0.05%).

**Table 3:** Physical characteristics of biscuits.

Samples	L*	a*	b*	Thickness (mm)	Diameter (mm)	a_w_
F0	37.92 ± 0.37 ^cd^	0.37 ± 0.67 ^de^	22.77 ± 1.09 ^bc^	5.63 ± 0.51 ^ab^	43.73 ± 2.02 ^a^	0.19 ± 0.01 ^cd^
F1	38.26 ± 0.23 ^cd^	−0.04 ± 0.09 ^de^	23.60 ± 0.46 ^bc^	5.15 ± 0.18 ^abc^	42.55 ± 0.60 ^a^	0.32 ± 0.00 ^a^
F2	39.96 ± 0.27 ^c^	5.05 ± 0.57 ^a^	24.69 ± 1.59 ^a^	6.12 ± 0.55 ^a^	34.64 ± 2.40 ^ab^	0.35 ± 0.02 ^a^
F3	35.61 ± 0.13 ^e^	2.56 ± 0.14 ^bc^	19.82 ± 0.88 ^c^	5.45 ± 0.12 ^ab^	42.63 ± 1.15 ^a^	0.27 ± 0.01 ^b^
F4	49.74 ± 1.04 ^a^	1.18 ± 0.59 ^cd^	31.07 ± 0.65 ^a^	4.20 ± 0.10 ^c^	37.52 ± 5.98 ^ab^	0.17 ± 0.00 ^d^
F5	36.84 ± 0.08 ^de^	3.60 ± 0.49 ^ab^	21.40 ± 0.20 ^bc^	5.56 ± 0.01 ^ab^	38.96 ± 2.15 ^ab^	0.26 ± 0.00 ^b^
F6	37.38 ± 0.37 ^de^	3.10 ± 0.17 ^b^	22.37 ± 0.10 ^bc^	5.35 ± 0.11 ^abc^	29.98 ± 0.04 ^b^	0.20 ± 0.00 ^cd^
F7	38.21 ± 0.17 ^cd^	5.09 ± 0.27 ^a^	22.99 ± 1.68 ^bc^	5.74 ± 0.40 ^ab^	42.63 ± 1.22 ^a^	0.22 ± 0.02 ^bcd^
F8	37.92 ± 0.37 ^cd^	−0.37 ± 0.67 ^de^	22.77 ± 1.09 ^bc^	5.38 ± 0.01 ^ab^	42.72 ± 0.31 ^a^	0.36 ± 0.02 ^a^
F9	46.75 ± 1.31 ^b^	−0.97 ± 0.24 ^e^	30.43 ± 0.78 ^a^	4.90 ± 0.27 ^bc^	31.44 ± 0.16 ^b^	0.22 ± 0.02 ^bc^

The values are expressed as the mean ± standard deviation. Different superscript letters after values mean significant difference (ANOVA, Tukey’s test, *p* < 0.05%).

**Table 4:** Textural characteristics of biscuits.

Sample	Breaking Force (N)	Fracturability (mm)	Hardness (N)
F0	0.72 ± 0.16 ^a^	1.06 ± 0.21 ^b^	21.85 ± 1.65 ^a^
F1	1.50 ± 0.02 ^a^	1.05 ± 0.11 ^b^	26.72 ± 1.15 ^a^
F2	0.86 ± 0.09 ^a^	2.35 ± 0.62 ^a^	24.76 ± 2.11 ^a^
F3	0.95 ± 0.00 ^a^	0.98 ± 0.21 ^b^	26.44 ± 3.84 ^a^
F4	0.60 ± 0.00 ^a^	0.64 ± 0.25 ^b^	21.94 ± 2.78 ^a^
F5	1.24 ± 0.00 ^a^	1.00 ± 0.17 ^b^	28.98 ± 2.17 ^a^
F6	1.02 ± 0.02 ^a^	0.93 ± 0.20 ^b^	26.73 ± 1.59 ^a^
F7	1.25 ± 0.10 ^a^	0.80 ± 0.05 ^b^	24.61 ± 2.35 ^a^
F8	1.35 ± 0.13 ^a^	0.80 ± 0.06 ^b^	33.19 ± 2.18 ^a^
F9	0.53 ± 0.28 ^a^	0.70 ± 0.17 ^b^	20.53 ± 1.88 ^a^

The values are expressed as the mean ± standard deviation. Different superscript letters after values mean significant difference (ANOVA, Tukey’s test, *p* < 0.05%).

**Table 5:** Compositional analysis of the biscuit samples.

Biscuit Samples	Moisture (%)	Ash (%)	Fat (%)	Protein (%)	Carbohydrates (%)	Fiber (%)	Energy (kcal/100 g)
F0	4.13 ± 0.01 ^a^	3.96 ± 0.05 ^ab^	25.10 ± 0.50 ^ab^	12.07 ± 0.18 ^de^	54.74 ± 0.53 ^c^	2.78 ± 1.75 ^a^	488 ± 5.30 ^c^
F1	4.13 ± 0.02 ^a^	3.42 ± 0.27 ^cd^	17.68 ± 0.33 ^b^	13.54 ± 0.19 ^ef^	61.23 ± 0.46 ^a^	3.33 ± 0.13 ^a^	452 ± 4.90 ^f^
F2	4.07 ± 0.01 ^ab^	3.66 ± 0.02 ^bcd^	30.51 ± 0.28 ^ab^	15.32 ± 0.04 ^c^	46.44 ± 0.36 ^f^	3.77 ± 0.01 ^a^	514 ± 4.20 ^a^
F3	4.09 ± 0.01 ^ab^	4.06 ± 0.04 ^a^	31.95 ± 0.46 ^ab^	16.13 ± 0.16 ^b^	43.77 ± 0.50 ^g^	5.34 ± 0.07 ^a^	516 ± 5.10 ^a^
F4	4.05 ± 0.00 ^b^	3.33 ± 0.10 ^d^	19.86 ± 0.20 ^ab^	12.32 ± 0.01 ^g^	60.44 ± 0.23 ^ab^	2.12 ± 0.09 ^a^	466 ± 4.40 ^e^
F5	4.08 ± 0.03 ^ab^	3.73 ± 0.01 ^abc^	24.74 ± 0.05 ^ab^	14.82 ± 0.27 ^d^	52.63 ± 0.35 ^cd^	3.79 ± 0.08 ^a^	485 ± 4.90 ^d^
F6	4.07 ± 0.00 ^ab^	3.68 ± 0.02 ^abcd^	21.91 ± 0.02 ^ab^	14.90 ± 0.20 ^h^	55.44 ± 0.20 ^bc^	4.54 ± 0.01 ^a^	469 ± 4.00 ^e^
F7	4.08 ± 0.03 ^ab^	3.71 ± 0.02 ^abcd^	27.90 ± 0.06 ^a^	14.63 ± 0.17 ^d^	49.68 ± 0.18 ^de^	4.76 ± 0.04 ^a^	499 ± 4.00 ^b^
F8	4.07 ± 0.01 ^ab^	3.75 ± 0.00 ^abc^	26.24 ± 1.99 ^ab^	16.93 ± 0.18 ^a^	49.01 ± 2.00 ^de^	4.11 ± 0.08 ^a^	492 ± 3.30 ^c^
F9	4.05 ± 0.03 ^b^	3.57 ± 0.02 ^cd^	22.42 ± 0.24 ^ab^	13.10 ± 0.32 ^f^	56.86 ± 0.42 ^bc^	3.31 ± 0.08 ^a^	475 ± 4.60 ^d^

The values are expressed as the mean ± standard deviation. Different superscript letters after values mean significant difference (ANOVA, Tukey’s test, *p* < 0.05%).

**Table 6:** Total phenolic content (TPC), total flavonoid content (TFC), and radical scavenging activity of the biscuits.

Samples	TPC (mg GAE/100 g)	Flavonoids (mg QE/100 g)	DPPH (mM Trolox/100 g)
F0	52.88 ± 1.00 ^j^	110.44 ± 1.38 ^i^	7.37 ± 0.07 ^c^
F1	93.04 ± 1.72 ^c^	234.90 ± 2.60 ^d^	7.19 ± 0.01 ^d^
F2	63.52 ± 3.06 ^i^	123.46 ± 2.38 ^h^	7.72 ± 0.01 ^b^
F3	82.28 ± 2.60 ^e^	129.34 ± 2.06 ^g^	8.06 ± 0.03 ^a^
F4	68.16 ± 3.16 ^g^	96.18 ± 2.30 ^j^	7.64 ± 0.01 ^b^
F5	83.52 ± 2.80 ^d^	168.64 ± 2.72 ^e^	7.64 ± 0.04 ^b^
F6	97.84 ± 1.60 ^b^	288.46 ± 1.00 ^c^	7.31 ± 0.03 ^cd^
F7	67.26 ± 1.60 ^h^	335.30 ± 2.52 ^a^	7.98 ± 0.01 ^a^
F8	80.54 ± 2.42 ^f^	139.50 ± 3.64 ^f^	7.45 ± 0.07 ^c^
F9	120.66 ± 3.00 ^a^	297.70 ± 3.00 ^b^	6.88 ± 0.01 ^e^

The values are expressed as the mean ± standard deviation. Different superscript letters after values mean significant difference (ANOVA, Tukey’s test, *p* < 0.05%).

## Data Availability

The original contributions presented in this study are included in the article; further inquiries can be directed to the corresponding author.

## References

[1] Zydenbos S., Humphrey-Taylor V., Wrigley C. (2004). Cookies, Biscuits and Crackers: The Diversity of Products. Encyclopedia of Grain Science.

[2] Benkadri S. (2010). Farines-Biscuits Infantiles sans Gluten Pour Enfants Coeliaques: Aptitude Technologique de Formules à Base de Riz–Légumes Secs. Master’s Thesis.

[3] Dayakar B. (2017). Technology Involved in Quality of Biscuits: Influence of Factors and Impact on Processing–A Critical Review. Int. J. Pure Appl. Biosci..

[4] Jia M., Yu Q., Chen J., He Z., Chen Y., Xie J., Nie S., Xie M. (2020). Physical quality and in vitro starch digestibility of biscuits as affected by addition of soluble dietary fiber from defatted rice bran. Food Hydrocoll..

[5] Nhouchi Z., Botosoa E.P., Karoui R. (2018). Critical assessment of formulation, processing and storage conditions on the quality of alveolar baked products determined by different analytical techniques: A review. Trends Food Sci. Technol..

[6] Pawłowska K., Kuligowski M., Jasińska-Kuligowska I., Kidoń M., Siger A., Rudzińska M., Nowak J. (2018). Effect of Replacing Cocoa Powder by Carob Powder in the Muffins on Sensory and Physicochemical Properties. Plant Foods Hum. Nutr..

[7] Alam M.A., Alam M.J., Hakim M.A., Huq A.K.O., Moktadir S.M.G. (2014). Development of Fiber Enriched Herbal Biscuits: A Preliminary Study on Sensory Evaluation and Chemical Composition. Int. J. Nutr. Food Sci..

[8] Mafukata Z.P., Bamidele O.P., Ramashia S.E., Mashau M.E. (2024). Nutritional composition, protein digestibility and consumer acceptability of sorghum-based *mahewu* enriched with *Moringa oleifera* leaf powder. Int. J. Food Sci. Technol..

[9] Songré-Ouattara L.T., Goubgou M., Savadogo A. (2017). Impact de l’emballage et de la durée de conservation sur la qualité nutritionnelle et microbiologique des biscuits de sorgho enrichis au moringa et à la spiruline. J. Appl. Biosci..

[10] Bukonja S., Tomić J., Pestorić M., Maravić N., Despotović S., Tomičić Z., Kiprovski B., Pantelić N.Đ. (2025). Exploring Sorghum Flour as a Sustainable Ingredient in Gluten-Free Cookie Production. Foods.

[11] Hoyos B.E., Johnson J.B., Mani J.S., Batley R.J., Trotter T., Bhattarai S.P., Naiker M. (2024). Biochemical Characterisation of the Northern Australian-Grown Black Sesame as a Source of Bioactive Compounds. Master’s Thesis.

[12] Chang Y.-L., Qin Z., Jia H.-J., Wang R., Liu H.-M., Mei H.-X., Duan Y.-H., Zhang S.-Z. (2024). Comparison of the chemical composition of non-shattering and shattering sesame varieties grown in the Huang-Huai region of China. J. Food Compos. Anal..

[13] Sulastri E., Zubair M.S., Anas N.I., Abidin S., Hardani R., Yulianti R. (2018). Total Phenolic, Total Flavonoid, Quercetin Content and Antioxidant Activity of Standardized Extract of *Moringa oleifera* Leaf from Regions with Different Elevation. Pharmacogn. J..

[14] El-Fadl S.A., Osman A., Al-Zohairy A.M., Dahab A.A., El Kheir Z.A.A. (2020). Assessment of total phenolic, flavonoid content, antioxidant potential and hplc profile of three moringa species leaf extracts. Sci. J. Flowers Ornam. Plants.

[15] Fombang E.N., Nobossé P., Mbofung C.M.F., Singh D. (2021). Impact of post harvest treatment on antioxidant activity and phenolic profile of *Moringa oleifera* lam leaves. Food Prod. Process. Nutr..

[16] Scheffe H. (2018). Experiments with Mixtures. J. R. Stat. Soc. Ser. B (Methodol.).

[17] Yildiz S., Karakuş E., Öztürk S. (2023). D-Optimal mixture design approach in the production of cookies enriched with dietary fiber sources such as lentil flour, banana fruit and banana peel powder. GIDA/J. Food.

[18] Mouafo H.T., Matuekam A.D., Petagou I.L., Ngeudjo M.W., Baomog A.M.B., Ntsama P.M., Medoua G.N. (2024). Formulation of nutritious and functional meal-based biscuits from mixture of soybean, papaya fruit pulp, and baobab fruit pulp flours. Heliyon.

[19] Dabo R., Oboulbiga E.B., Semde Z., Kanté-Traoré H., Banhoro O., Compaoré-Sérémé D., Diarra S., Douamba Z., Ouédraogo B., Tapsoba F.W.-B. (2024). Technological Aptitude and Acceptability of Four Sesame Varieties from Burkina Faso in Pastes and Biscuits. Int. J. Food Sci. Agric..

[20] Inglett G.E., Chen D., Liu S.X. (2015). Physical properties of gluten-free sugar cookies made from amaranth–oat composites. LWT.

[21] Granato D., Masson M.L. (2010). Instrumental color and sensory acceptance of soy-based emulsions: A response surface approach. Food Sci. Technol..

[22] Luca M.I., Ungureanu-Iuga M., Mironeasa S. (2022). Carrot Pomace Characterization for Application in Cereal-Based Products. Appl. Sci..

[23] Minekus M., Alminger M., Alvito P., Ballance S., Bohn T., Bourlieu C., Carrière F., Boutrou R., Corredig M., Dupont D. (2014). A Standardised Static in Vitro Digestion Method Suitable for Food—An International Consensus. Food Funct..

[24] Vongsak B., Sithisarn P., Mangmool S., Thongpraditchote S., Wongkrajang Y., Gritsanapan W. (2013). Maximizing total phenolics, total flavonoids contents and antioxidant activity of *Moringa oleifera* leaf extract by the appropriate extraction method. Ind. Crop. Prod..

[25] Pakade V., Cukrowska E., Chimuka L. (2013). Comparison of antioxidant activity of *Moringa oleifera* and selected vegetables in South Africa. S. Afr. J. Sci..

[26] Boukid F., Diantom A., Corte R., Curti E., Carini E., Vittadini E. (2020). Structured fat–water–fiber systems as fat substitutes in shortbread formulation: Modulation of dough characteristics following a multiscale approach. Eur. Food Res. Technol..

[27] Cervini M., Frustace A., Garrido G.D., Rocchetti G., Giuberti G. (2021). Nutritional, physical and sensory characteristics of gluten-free biscuits incorporated with a novel resistant starch ingredient. Heliyon.

[28] Adedara O.A., Taylor J.R.N. (2021). Roles of Protein, Starch and Sugar in the Texture of Sorghum Biscuits. LWT.

[29] Giuberti G., Bresciani A., Cervini M., Frustace A., Marti A. (2021). *Moringa oleifera* L. leaf powder as ingredient in gluten-free biscuits: Nutritional and physicochemical characteristics. Eur. Food Res. Technol..

[30] Jonkers N., van Dommelen J.A.W., Geers M.G.D. (2022). Intrinsic mechanical properties of food in relation to texture parameters. Mech. Time-Depend. Mater..

[31] Guiné R.P.F. (2022). Textural Properties of Bakery Products: A Review of Instrumental and Sensory Evaluation Studies. Appl. Sci..

[32] Jan R., Saxena D., Singh S. (2016). Physico-chemical, textural, sensory and antioxidant characteristics of gluten—Free cookies made from raw and germinated Chenopodium (*Chenopodium album*) flour. LWT.

[33] Ayo-Omogie H. (2023). Unripe Banana and Defatted Sesame Seed Flours Improve Nutritional Profile, Dietary Fibre and Functional Properties of Gluten-Free Sorghum Cookies. Food Prod. Process. Nutr..

[34] Rabie M.M., Ibrahim F.Y., Youssif M.R.G., Ezz El-Ragal N.M. (2020). Effect of *Moringa oleifera* Leaves and Seeds Powder Supplementation on Quality Characteristics of Cookies. J. Food Dairy Sci..

[35] Zouari R., Besbes S., Ellouze-Chaabouni S., Ghribi-Aydi D. (2016). Cookies from composite wheat–sesame peels flours: Dough quality and effect of Bacillus subtilis SPB1 biosurfactant addition. Food Chem..

[36] Alemu T. (2022). Texture Profile and Design of Food Product. J. Agri. Horti. Res..

[37] Sengev A.I., Abu J.O., Gernah D.I. (2013). Effect of *Moringa Oleifera* Leaf Powder Supplementation on Some Quality Characteristics of Wheat Bread. Food Nutr. Sci..

[38] Caporizzi R., Derossi A., Devahastin S., Severini C. (2025). Boosting the role of complex food structure on oral breakdown and sweetness perception by digitally designed and 3D printed biscuits. J. Food Eng..

[39] Ghendov-Mosanu A., Ropciuc S., Dabija A., Saitan O., Boestean O., Paiu S., Rumeus I., Leatamborg S., Lupascu G., Codină G.G. (2025). Effect of Brewers’ Spent Grain Addition to a Fermented Form on Dough Rheological Properties from Different Triticale Flour Cultivars. Foods.

[40] Tan J.-M., Li B., Han S.-Y., Wu H. (2023). Use of a compound modifier to retard the quality deterioration of frozen dough and its steamed bread. Food Res. Int..

[41] Azar G., Demirkesen I. (2023). Linear and Non Linear Rheological Properties of Gluten Free Dough Systems Probed by Fundamental Methods. Food Eng. Rev..

[42] Huschka B., Challacombe C., Marangoni A.G., Seetharaman K. (2011). Comparison of Oil, Shortening, and a Structured Shortening on Wheat Dough Rheology and Starch Pasting Properties. Cereal Chem..

[43] Dreese P.C., Faubion J.M., Hoseney R.C. (1988). Dynamic Rheological Properties of Flour, Gluten, and Gluten-Starch Doughs. I. Tempe-rature-Dependent Changes During Heating. Cereal Chem..

[44] Codină G.G., Dabija A., Oroian M. (2019). Prediction of Pasting Properties of Dough from Mixolab Measurements Using Artificial Neuronal Networks. Foods.

[45] Salvador A., Sanz T., Fiszman S.M. (2006). Dynamic Rheological Characteristics of Wheat Flour-Water Doughs. Effect of Adding NaCl, Sucrose and Yeast. Food Hydrocoll..

[46] Nour V., Blejan A.M., Codină G.G. (2025). Use of Bilberry and Blackcurrant Pomace Powders as Functional Ingredients in Cookies. Appl. Sci..

[47] Culețu A., Mohan G., Duță D.E. (2020). Rheological Characterization of the Dough with Added Dietary Fiber by Rheometer: A Review. Bull. Univ. Agric. Sci. Veter-Med. Cluj-Napoca. Food Sci. Technol..

[48] Pilling S., Andrade D.P.P., da Silveira E.F., Rothard H., Domaracka A., Boduch P. (2012). Formation of unsaturated hydrocarbons in interstellar ice analogues by cosmic rays. Mon. Not. R. Astron. Soc..

[49] Sipaut C.S., Dayou J. (2019). *In situ* FTIR analysis in determining possible chemical reactions for peroxide crosslinked LDPE in the presence of triallylcyanurate. Funct. Compos. Struct..

[50] Adebiyi J.A., Obadina A.O., Mulaba-Bafubiandi A.F., Adebo O.A., Kayitesi E. (2016). Effect of fermentation and malting on the microstructure and selected physicochemical properties of pearl millet (*Pennisetum glaucum*) flour and biscuit. J. Cereal Sci..

[51] Nicy A.B., Velayutham P., Ganesan P., Shakila R.J. (2021). Functional and structural characteristics of chocolate flavoured cake incorporated with surimi powder from Nemipterus species. J. Food Sci. Technol..

[52] Zarroug Y., Nasri S., Sfayhi D., Aziza Z.K., Eya F., Mohamed K. (2021). Formulation de Biscuits Enrichis Par La Farine Des Graines de *Vicia narbonensis* L. Ann. l’INRAT.

[53] Karaca Açarı İ. (2021). Determination of the Chemical Structure of Diet Biscuits with Modern Enstrumental Techniques. Cumhur. Sci. J..

[54] Srivastava S., Genitha T.R., Yadav V. (2012). Preparation and Quality Evaluation of Flour and Biscuit from Sweet Potato. J. Food Process. Technol..

[55] Ifediba D.I., Egbuna H.I. (2019). Proximate Composition and Organoleptic Properties of Whole Wheat Biscuit Fortified with Moringa (*Moringa Oleifera*) Leaf Powder. Int. J. Sci. Res. Publ. (IJSRP).

[56] Kaur A., Kumar K., Dhaliwal H.S. (2020). Physico-chemical characterization and utilization of finger millet (*Eleusine coracana* L.) cultivars for the preparation of biscuits. J. Food Process. Preserv..

[57] Zarroug Y., Sriti J., Sfayhi D., Slimi B., Allouch W., Zayani K., Hammami K., Sowalhia M., Kharrat M. (2021). Effect of addition of Tunisian *Zizyphus lotus* L. Fruits on nutritional and sensory qualities of cookies. Ital. J. Food Sci..

[58] Bhuker A., Malik A., Punia H., McGill C., Sofkova-Bobcheva S., Mor V.S., Singh N., Ahmad A., Mansoor S. (2023). Probing the Phytochemical Composition and Antioxidant Activity of *Moringa oleifera* under Ideal Germination Conditions. Plants.

[59] Diatta-Holgate E., Hugghis E., Weil C., Faye J.M., Danquah A., Diatta C., Tongoona P., Danquah E.Y., Cisse N., Tuinstra M.R. (2022). Natural variability for protein digestibility and grain quality traits in a West African Sorghum Association Panel. J. Cereal Sci..

[60] Anyiam P.N., Phongthai S., Sai-Ut S., Kingwascharapong P., Jung Y.H., Zhang W., Rawdkuen S. (2025). Nutritional Components and Digestibility Profiles of Some Potential Plant-Based Protein Sources. Foods.

[61] Gaudichon C., Benhaddou S. (2024). Physiologie de la Digestion des Protéines et de l’absorption des Acides Aminés et Déterminants de la Digestibilité Protéique (Physiology of Protein Digestion and Amino Acid Absorption and Factors Influencing Protein Digestibility). Médecine Mal. Métaboliques.

